# Genomic prediction and association mapping of maize grain yield in multi-environment trials based on reaction norm models

**DOI:** 10.3389/fgene.2023.1221751

**Published:** 2023-08-31

**Authors:** Seth A. Tolley, Luiz F. Brito, Diane R. Wang, Mitchell R. Tuinstra

**Affiliations:** ^1^ Department of Agronomy, Purdue University, West Lafayette, IN, United States; ^2^ Department of Animal Sciences, Purdue University, West Lafayette, IN, United States

**Keywords:** genomic prediction, genome-wide association study, Genomes to Fields, multi-environment trial, genotype-by-environment interaction

## Abstract

Genotype-by-environment interaction (GEI) is among the greatest challenges for maize breeding programs. Strong GEI limits both the prediction of genotype performance across variable environmental conditions and the identification of genomic regions associated with grain yield. Incorporating GEI into yield prediction models has been shown to improve prediction accuracy of yield; nevertheless, more work is needed to further understand this complex interaction across populations and environments. The main objectives of this study were to: 1) assess GEI in maize grain yield based on reaction norm models and predict hybrid performance across a gradient of environmental (EG) conditions and 2) perform a genome-wide association study (GWAS) and post-GWAS analyses for maize grain yield using data from 2014 to 2017 of the Genomes to Fields initiative hybrid trial. After quality control, 2,126 hybrids with genotypic and phenotypic data were assessed across 86 environments representing combinations of locations and years, although not all hybrids were evaluated in all environments. Heritability was greater in higher-yielding environments due to an increase in genetic variability in these environments in comparison to the low-yielding environments. GWAS was carried out for yield and five single nucleotide polymorphisms (SNPs) with the highest magnitude of effect were selected in each environment for follow-up analyses. Many candidate genes in proximity of selected SNPs have been previously reported with roles in stress response. Genomic prediction was performed to assess prediction accuracy of previously tested or untested hybrids in environments from a new growing season. Prediction accuracy was 0.34 for cross validation across years (CV0-Predicted EG) and 0.21 for cross validation across years with only untested hybrids (CV00-Predicted EG) when compared to Best Linear Unbiased Prediction (BLUPs) that did not utilize genotypic or environmental relationships. Prediction accuracy improved to 0.80 (CV0-Predicted EG) and 0.60 (CV00-Predicted EG) when compared to the whole-dataset model that used the genomic relationships and the environmental gradient of all environments in the study. These results identify regions of the genome for future selection to improve yield and a methodology to increase the number of hybrids evaluated across locations of a multi-environment trial through genomic prediction.

## 1 Introduction

Maize is among the most important crops globally and is the most economically important crop in the United States where the average yield was 10.8 Mg ha^−1^ and total production of 360 million tonnes valued at $59.6 billion dollars in 2020 (USDA NASS). Extensive private sector hybrid development and testing along with improved agronomic and management practices have contributed to sustained yield improvement in the United States (Duvick et al., 2004). Nevertheless, yield performance is largely environment-dependent where change in ranks or magnitude among hybrids is common across environments. This genotype-by-environment interaction (GEI) necessitates breeding programs carry out costly multi-environment trials (MET) to optimize hybrid development and placement (Cooper et al., 2016). Understanding the mechanisms that give rise to GEI and predicting yield performance across environments is a major goal of maize breeding programs.

Genome-wide association studies (GWAS) are an important tool to uncover the complex genetic structure of quantitative traits, such as grain yield, which have many small-effect quantitative trait loci (QTL) involved. GWAS utilizes dense genomic data to identify associations between genomic markers and phenotypic traits. Causal markers or markers in linkage disequilibrium (LD) with QTL are detected as being associated with the phenotypes they control (Yu and Buckler, 2006). GWAS have previously been used in maize to detect associations with yield and yield components (Millet et al., 2016; Ma & Cao et al., 2021), drought and heat stress tolerance (Xue et al., 2013; Yuan et al., 2019), nutrient stress tolerance (Ndlovu et al., 2022), and many other traits. These marker-trait associations are dependent on the environment where a given QTL could be important in one environment and not in another environment (Millet et al., 2016; Yuan et al., 2019). Nevertheless, these studies are often performed in few environments, which limits the inference to a given region or type of environment. Assessing single nucleotide polymorphisms (SNPs) with widespread utility compared to those that are environment dependent could be beneficial to better understand the QTL-by-environment relationships.

Marker-assisted selection (MAS) where hybrids are selected based on major QTL has been an important breeding technique for traits controlled by few, major effect QTL. Genomic prediction (GP) that uses high-density markers to predict hybrid yield performance has been shown to improve genetic gain approximately three-fold compared to selection based on single or few genetic markers (MAS) in quantitative traits (Heffner et al., 2010). A training dataset of hybrids with phenotypic and genotypic data is used to model hybrid performance in GP (Meuwissen et al., 2001). This model built on the training dataset is applied to predict performance of untested hybrids in the tested environments, tested hybrids in an untested environment, or untested hybrids in an untested environment. Accuracy is maximized when the hybrids and environments in the training dataset resemble those being predicted in the testing dataset (Sapkota et al., 2020). Various models have been developed for genomic prediction. The Genomic Best Linear Unbiased Prediction (GBLUP) model, which uses a relationship matrix based on pedigree or genomic data, is widely used due to the computational efficiency and prediction equivalence when compared to more complicated models (Heslot et al., 2012; Crossa et al., 2014).

Previous work has demonstrated that genomic prediction models are improved by incorporating GEI analysis (Burgueño et al., 2012; Jarquín et al., 2014). One approach has been to treat grain yield from each environment as independent traits and use a multi-trait model (Burgueño et al., 2012; Montesinos-López et al., 2016). More recent studies have modeled grain yield across environments as a single trait in a reaction norm model (RNM) where the environments are described based on continuous environmental gradients such as weather and soil characteristics (Jarquín et al., 2014; Pérez-Rodríguez et al., 2015; Jarquín et al., 2017; Pérez-Rodríguez et al., 2017; Sukumaran et al., 2018; Monteverde et al., 2019; Rincent et al., 2019; De Los Campos et al., 2020; Westheus et al., 2021). Jarquín et al. (2014) reported a large increase in GP prediction accuracy where GEI were accounted for using continuous environmental characteristics. Relatively less work has been done where environments are described based on their estimated merit (Finlay and Wilkinson, 1963; Malosetti et al., 2013; Gage et al., 2017; Chen et al., 2021).

The Genomes to Fields (G2F) initiative (www.genomes2fields.org) is a large-scale partnership between the public and private sector to organize a MET where inbred and hybrid maize genotypes are evaluated across a range of environments across North America. Phenotypic, genotypic, and environmental data collected in each site are publicly available (Falcon et al., 2020; McFarland et al., 2020). The main objectives of this study were to 1) characterize the heritability and genetic correlation of hybrid maize yield in 86 environments, 2) calculate genomic estimated breeding value (GEBV) of all hybrids across a gradient of environments, 3) evaluate which SNPs have the greatest magnitude of effect in each environment, and 4) assess the accuracy of genomic prediction for maize grain yield using RNM.

## 2 Materials and methods

### 2.1 Phenotypic data

Phenotypic and genotypic data from 2014 to 2017 of the G2F initiative was obtained from Cyverse (G2F Consortium, 2019). Phenotypic information was collected in MET across North America through a collaboration of 67 principal investigators and sponsors across 18 universities and federal agencies (numbers from 2022) (Supplementary Table S1; Supplementary Figure S1). Experimental design generally followed two-row plots organized in a randomized complete block design with two replications. From 2014 to 2017, there were 59,416 plots in 108 environments (unique locations and/or years) and 2,521 hybrids. Grain yield was evaluated in 105 of the 108 environments. Plots with a grain yield greater or less than three standard deviations from the mean of a given environment were removed. Spatial correction within each environment where row and range information was available was performed using the R package “SpATS” (Rodríguez-Álvarez et al., 2018). Repeatability improved after the spatial correction in 97 of the 105 environments with an average increase in repeatability of 0.18 (Supplementary Figure S2). Five environments had a repeatability less than 0.1 and were removed from the analysis. Ten environments were removed from consideration as latitude and longitude information was not available. Seven environments were removed from consideration due to problematic phenotypic data collection indicated by collaborators in the metadata. Hybrids without genomic information were removed from analyses leaving 2,126 hybrids from 39,305 plots in 86 environments.

### 2.2 Genotypic data

Genotyping-by-sequencing (GBS) data for 1,577 inbred lines with 945,574 SNPs is publicly available at Cyverse (G2F Consortium, 2019). Gage et al. (2017) further described the GBS procedure used in the G2F initiative. A total of 842 of the 1,577 inbred lines were used in the G2F hybrid experiment. Quality control on the genotypic dataset of the inbred lines used in the study was performed with TASSEL 5 (Bradbury et al., 2007). Heterozygous SNPs were made unknown as these would be unlikely in inbred lines. Minor SNP states beyond the most common biallelic pair were removed from consideration. These were performed to remove SNPs possibly subject to sequencing error. SNPs with a minor allele frequency less than 1% were removed from analyses leaving 396,215 SNPs. SNPs missing in more than 5% of inbred lines were removed leaving 100,878 SNPs. Missing calls at these SNPs were imputed using Beagle v5.4 (Brown et al., 2021). Beagle has previously been shown to be an effective strategy for imputing genotype data in maize (de Oliveira et al., 2020). From this imputed inbred line dataset, hybrid genotypes were created using TASSEL 5 (Bradbury et al., 2007) and the SNPs were coded based on the number of major alleles at each locus (0, 1, or 2). After quality control, there were 2,143 hybrids with 100,878 SNPs. Of the 2,143 hybrids, 2,126 remained after the phenotypic quality control. For the 100,878 SNPs, the median distance between adjacent SNPs was 63 base pairs (bp) while the average distance between adjacent SNPs was 20,844 bp.

Linkage disequilibrium (LD) was calculated using the hybrid dataset for the 2,126 hybrids with 100,878 SNPs. LD was calculated using a sliding window of 100 SNPs using PLINK v1.9 (Purcell et al., 2007) (Supplementary Figure S3).

### 2.3 Reaction norm models

Reaction norm models were used to model grain yield of the 2,126 hybrids in 86 environments. The merit of an environment was estimated as the fixed effect of environment in a BLUP model to mitigate differences in average yield performance due to different hybrids evaluated in different environments. The environmental gradient (EG) was estimated as β using the BLUP model implemented in R package “lme4” (Bates et al., 2015) in Eq. 1.

y=Xβ+Zg+ε (1)

where ***y*** is the vector of phenotypic measurements, **X** is a design matrix associating the phenotypic measurement to the environment (combination of location and year), β is the vector of the fixed effect of the environment, **Z** is the design matrix associating the phenotypic data and the hybrid identification, **g** is the vector of BLUPs with $g∼N\left(0,I\sigma_{g}^{2}\right)$ where $\sigma_{g}^{2}$ is the genetic variance and **I** represents an identity matrix, and ε is the vector of random residuals with $\varepsilon \;∼N\left(0,I\sigma_{\varepsilon }^{2}\right)$ where $\sigma_{\varepsilon }^{2}$ is residual variance and **I** is an identity matrix. This model utilized an identity rather than genomic relationship matrix as the goal was to estimate β . The EG was scaled to range from −1 to 1 using Eq. 2 (Coelho et al., 2020).

$$\theta_{i}=-1+2\left[\left(\beta_{i}-\beta_{\min}\right)/\left(\beta_{\max}-\beta_{\min}\right)\right]$$ (2)

where $\theta_{i}$ is the standardized EG for environment *i*, $\beta_{i}$ is the coefficient of environment *i,* and $\beta_{\min}$ and $\beta_{\max}$ are the minimum and maximum values in the vector of β , respectively.

Genomic analyses were performed through GBLUP based on RNM. The 2^nd^ order Legendre orthogonal polynomial RNM model is defined in Eq. 3.

$$y_{ij}=Env+b_{m}{\hat{\theta }}_{i}+\sum \left(n_{0_{j}}+n_{1_{j}}{\hat{\theta }}_{i}+n_{2_{j}}{{\hat{\theta }}_{i}}^{2}\right)+\varepsilon_{ij}$$ (3)

where $y_{ij}$ is the phenotypic measurement for the *j*th hybrid in the *i*th EG coefficient, **Env** is the vector of the fixed effect of the location-year combination, *b*_*m*_ is the *m*th fixed regression coefficient for the average curve of the population, ${\hat{\theta }}_{i}$ is the standardized EG for environment *i*, $n_{0_{j}},n_{1_{j}},and\;n_{2_{j}}$ are the intercept, slope, and polynomial of the *j*th hybrid regressed on ${\hat{\theta }}_{i}$ for random effects *n* (*n* $\epsilon \left\{a,be\right\})$ where *a* is the additive genetic effect and *be* is the broad environment effect, $\varepsilon_{ij}$ is the random residual of the *j*th hybrid in the *i*th EG. The covariance structure for the additive genetic variance of the intercept, slope, and polynomial were as follows:

$$\left[\begin{array}{c} a_{0}\\ a_{1}\\ a_{2} \end{array}\right]\;∼\;N\;\left(0,G\;⊗\left[\begin{array}{ccc} \sigma_{a_{0}}^{2} & \sigma_{a_{0}a_{1}} & \sigma_{a_{0}a_{2}}\\ \sigma_{a_{0}a_{1}} & \sigma_{a_{1}}^{2} & \sigma_{a_{1}a_{2}}\\ \sigma_{a_{0}a_{2}} & \sigma_{a_{1}a_{2}} & \sigma_{a_{2}}^{2} \end{array}\right]\right)$$

and

$$\left[\begin{array}{c} {be}_{0}\\ {be}_{1}\\ {be}_{2} \end{array}\right]\;∼N\;\left(0,I\;⊗\left[\begin{array}{ccc} \sigma_{{be}_{0}}^{2} & \sigma_{{be}_{0}{be}_{1}} & \sigma_{{be}_{0}{be}_{2}}\\ \sigma_{{be}_{0}{be}_{1}} & \sigma_{{be}_{1}}^{2} & \sigma_{{be}_{1}{be}_{2}}\\ \sigma_{{be}_{0}{be}_{2}} & \sigma_{{be}_{1}{be}_{2}} & \sigma_{{be}_{2}}^{2} \end{array}\right]\right)$$

where $\sigma_{n_{0}}^{2}$ , $\sigma_{n_{1}}^{2}$ , and $\sigma_{n_{2}}^{2}$ are the variance of random effects $n_{0_{j}},n_{1_{j}},and\;n_{2_{j}}$ , respectively. The off-diagonal elements of the matrices are the covariances of the random effects. **G** is the genomic relationship matrix (GRM) using the first method defined in VanRaden (2008). **I** is an identity matrix.

The *be* effect in this context is similar to the permanent effect in random regression models. Differently from a traditional random regression model (longitudinal data for the same individual over time), the repeated records for the same hybrid are spatially-repeated measurements (i.e., in different environments). Model performance was compared with and without *be* using AIC (data not shown). This effect was found to reduce AIC and was considered throughout the remainder of the study. The reasoning for this effect is that *be* is capturing variation due to environmental effects on the hybrid’s phenotype caused by the high degree of similarity between the environments adjacent on the environmental gradient. Nevertheless, *be* could be capturing variance caused by non-additive genetic or systematic effects.

Multiple models were compared for performance using AIC including a linear and a 2nd order polynomial model with homogeneous or heterogeneous residual variance. The linear model was like the previously defined model in Eq. 3, but the effects related to the 2nd order polynomial coefficient were removed. Models were evaluated with homogeneous or heterogeneous variances. For the heterogeneous model, a different residual variance was used for each environment. The residual variance for each environment was exponentially regressed against the EG effect for that environment as in Eq. 4.

$$\sigma_{\varepsilon_{i}}^{2}=\exp\left(d_{0}+d_{1}{\hat{\theta }}_{i}\right)$$ (4)

where $d_{0}\;and\;d_{1}$ are the intercept and slope of the regression for heterogeneous residual variance (Foulley and Quaas, 1995; Chen et al., 2021). Models were compared based on AIC values and the optimal model was used for further analyses. Variance components were assessed using average-information restricted maximum likelihood implemented in the BLUPF90+ software (Misztal et al., 2002; Misztal et al., 2014).

### 2.4 Estimation of genetic parameters

The genetic covariance matrix ( Σ) for all environments is described in Eq. 5 and previously defined in (Oliveira et al., 2019; Moreira et al., 2021).

$$\Sigma =TCT^{\prime }$$ (5)

where **T** is the matrix of covariates including the intercept, slope, and polynomial for each environment. **C** is the genomic covariance matrix for the coefficients. This covariance matrix was used to define the narrow-sense heritability (h^2^) and genetic correlation of the environments in Eqs 6, 7.

$$h_{i}^{2}=\frac{{\hat{\sigma }}_{a_{i}}^{2}}{{\hat{\sigma }}_{a_{i}}^{2}+{\hat{\sigma }}_{\varepsilon_{i}}^{2}}$$ (6)

where ${\hat{\sigma }}_{a_{i}}^{2}$ is the estimated genetic variance for the *i*th environment and ${\hat{\sigma }}_{\varepsilon_{i}}^{2}$ is the estimated residual variance for the *i*th environment (in the heterogeneous model). The genetic correlation of yield across environments is defined in Eq. 7.

$$r_{{i,i}^{\prime }}=\frac{{\hat{\sigma }}_{a_{i,i^{\prime }}}}{\sqrt{{\hat{\sigma }}_{a_{i}}^{2}*\;{\hat{\sigma }}_{a_{i^{\prime }}}^{2}}}$$ (7)

where ${\hat{\sigma }}_{a_{i,i^{\prime }}}$ is the genetic covariance between the *i*th and the *i*'th environments, ${\hat{\sigma }}_{a_{i}}^{2}\;and\;{\hat{\sigma }}_{a_{i^{\prime }}}^{2}$ are the additive variances for the *i*th and the *i*'th environments, respectively. GEBVs are defined in Eq. 8.

$${\hat{GEBV}}_{j}=T{\hat{a}}_{j}$$ (8)

where ${\hat{a}}_{j}$ is the vector of predicted values for the intercept, slope, and polynomial for the *j*th hybrid. The predicted values, ${\hat{a}}_{j}$ , were estimated using BLUPF90+ (Aguilar et al., 2014).

### 2.5 Genome-wide association study

Additive SNP effects for GWAS were derived from GEBVs for the regression coefficients according to Eq. 9 (Wang et al., 2012; Moreira et al., 2021).

$${\hat{u}}_{j}=IZ^{\prime }{\left(ZIZ\right)}^{-1}{\hat{a}}_{j}$$ (9)

where ${\hat{u}}_{j}$ is the estimate for the SNP effect for the *j*th RNM coefficient, **Z** is the genomic information for all hybrids, **I** is an identity matrix, and ${\hat{a}}_{j}$ is the estimated GEBV for the *j*th RNM coefficient. SNP effects were determined using POSTGSF90 (Misztal et al., 2014). SNP effects were obtained for each environment according to Eq. 10.

$${\hat{SNP}}_{s}=T{\hat{u}}_{s}$$ (10)

where ${\hat{SNP}}_{s}$ is a vector containing the SNP effects for the *s*th SNP in each environment, ${\hat{u}}_{s}$ is the vector of SNP effects for each RNM coefficient for the *s*th SNP.

Candidate genes were evaluated for the top five SNPs with the highest magnitude of effect in each environment (Oliveira et al., 2019; Moreira et al., 2021). Candidate genes were explored that were ±1 kb from the selected SNPs using the Maize B73 v4 reference genome in https://www.maizegdb.org. The search window was defined based on the distance at which average LD decayed to 0.2, which was around 1 kb (Supplementary Figure S3). Gene function was predicted for each candidate gene in the UniProt database (https://www.uniprot.org/).

ANOVA was performed to determine if there were significant differences between the yield performance of hybrids that were homozygous for the minor allele, heterozygous, and homozygous for the major allele for the selected SNPs in each environment which resulted in 1,806 comparisons (Supplementary Table S2). Where there were significant differences between the yields of the hybrids in ANOVA, least significant difference (LSD) test was implemented in R package “agricolae” (de Mendiburu, 2021) to determine which hybrids were significantly different at ρ < 0.05. Additional analysis was performed to identify SNPs with a favorable minor allele frequency as these have the greatest potential to improve yield when selected for in the population. To decrease bias in this analysis, data from an environment was removed where there were fewer than 10 hybrids homozygous for the minor allele, homozygous for the major allele, or heterozygous. Additionally, only environments greater than 0 on the EG were considered as yield was more heritable in these environments.

### 2.6 Genomic prediction of breeding values

Genomic prediction was performed to simulate two breeding program scenarios: previously tested hybrids from another environment could be included in the training dataset to predict performance in an untested environment (CV0) or untested hybrids evaluated in an untested year (CV00) and were previously described in Jarquín et al. (2017) and Westhues et al. (2021). In each of these scenarios, 3 years were used to train a model and a single year was used in testing. Variance components were estimated using the training set and used to predict GEBV of the testing set.

The environmental merit, an environments placement along the environmental gradient, is unknown where yield has not been evaluated, as in the testing set of genomic prediction. To assess the environmental merit, environment-specific weather and soil data from public application programming interfaces (APIs) were used in a Random Forest model using the R packages “caret” (Kuhn et al., 2008) and “randomForest” (Liaw and Wiener, 2002). The environmental merit of the testing year of GP was predicted using a model built on the training data (other 3 years of data) where the weather and soil data was associated to the known environmental merit. The variables included in the model are described in Table 1. Weather data was acquired using the R package “nasapower” (Sparks et al., 2018) and was compiled by month from April to October (growing season). Various functions were used to summarize the weather data monthly. Total was used for precipitation and photosynthetically active radiation. Maximum temperature per day and wind speed per day were summarized monthly using the maximum of these values. Minimum daily temperature and the dew point were summarized using the minimum of these values. All other weather variables were summarized as the mean value throughout the month. Soil data was obtained from the National Resources Conservation Service (NRCS) using the R package “soilDB” (Beaudette et al., 2022). The variables used in the model were soil organic carbon and nitrogen content at depths of 0–5 cm, 5–15 cm, and 15–30 cm. The hyperparameters mtry and ntree were optimized using 10-fold cross validation within the training dataset.

**TABLE 1:** Environmental characteristics from r packages “nasapower” (Sparks et al., 2018) and “soilDB” (Beaudette et al., 2022).

Characteristic ID	Unit	Source	Function	Description
ALLSKY_SFC_PAR_TOT	W/m ^ 2	nasapower	Total	All Sky Surface PAR Total
T2MWET	cg/kg	nasapower	Mean	Wet Bulb Temperature at 2 m
QV2M	g/kg	nasapower	Mean	Specific Humidity at 2 m
RH2M	%	nasapower	Mean	Relative Humidity at 2 m
T2M_MAX	C	nasapower	Max	Temperature at 2 m Maximum
PS	kPa	nasapower	Mean	Surface Pressure
T2MDEW	C	nasapower	Minimum	Dew/Frost Point at 2 m
WS2M	m/s	nasapower	Max	Wind Speed at 2 m
T2M_MIN	C	nasapower	Minimum	Temperature at 2 m Minimum
T2M	C	nasapower	Mean	Temperature at 2 m
PRECTOTCORR	mm/day	nasapower	Total	Precipitation Corrected
Sand	g/kg	NRCS	Mean	Proportion of sand particles (>0.05 mm) in the fine earth fraction
Silt	g/kg	NRCS	Mean	Proportion of silt particles (= 0.002 mm and = 0.05 mm) in the fine earth fraction
Clay	g/kg	NRCS	Mean	Proportion of clay particles (<0.002 mm) in the fine earth fraction
SOC	dg/kg	NRCS	Mean	Soil organic carbon content in the fine earth fraction
Nitrogen	cg/kg	NRCS	Mean	Total nitrogen (N)

Characteristic ID, unit, and description come directly from the source. Function describes how the characteristic was combined in each month. Soil characteristics were gathered from intervals of 0–5 cm, 5–15 cm, and 15–30 cm.

Additionally, GP was performed where the known environmental merit was used in the environmental gradient to evaluate the impact of the discrepancy between the predicted and known environmental merits and was labeled CV0-Known EG.

GEBVs from GP were either associated to single-environment BLUPs or to the GEBV of the whole-dataset model where all data was used in training. Prediction accuracy was assessed using Pearson’s correlation of these values in each environment. Bias (*b*_*1*_) was evaluated for each environment using a linear regression model (BLUP = *b*_*0*_ *+ b*_1_*GEBV_*testing*_) or (GEBV_*whole*_ = *b*_*0*_ *+ b*_1_*GEBV_*testing*_). Bias was centered around 0 by subtracting one from the regression coefficient.

### 2.7 Additional information

Data analyses were performed using the R software v4.2 (R Core Team, 2022). Data visualization was performed using the R package “ggplot2” (Wickham, 2016). R code used for data preparation and visualization and BLUPF90 configuration files used in analyses are at the Purdue University Research Repository (Tuinstra and Tolley, 2023).

## 3 Results

### 3.1 Environmental characterization

**FIGURE 1:**
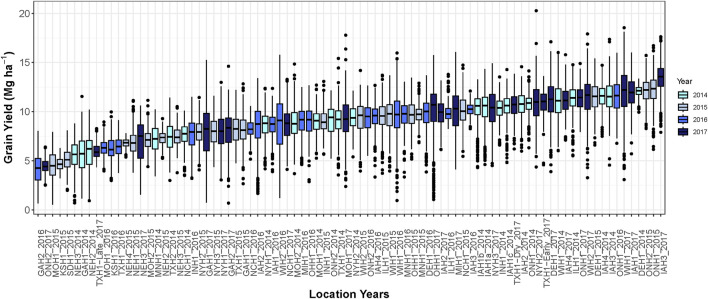
Boxplot demonstrating hybrid yield performance in each of the 86 environments. Different colors represent environments grown in different growing seasons.

The average grain yield among environments ranged from 4.2 to 13.5 Mg ha^−1^ (Figure 1). The lowest average grain yield was environment Watkinsville, GA in 2016 and the highest grain yield was Keystone, IA in 2017. The average grain yield was 9.3, 8.4, 8.9, and 10.2 Mg ha^−1^ in 2014, 2015, 2016, and 2017, respectively.

**FIGURE 2:**
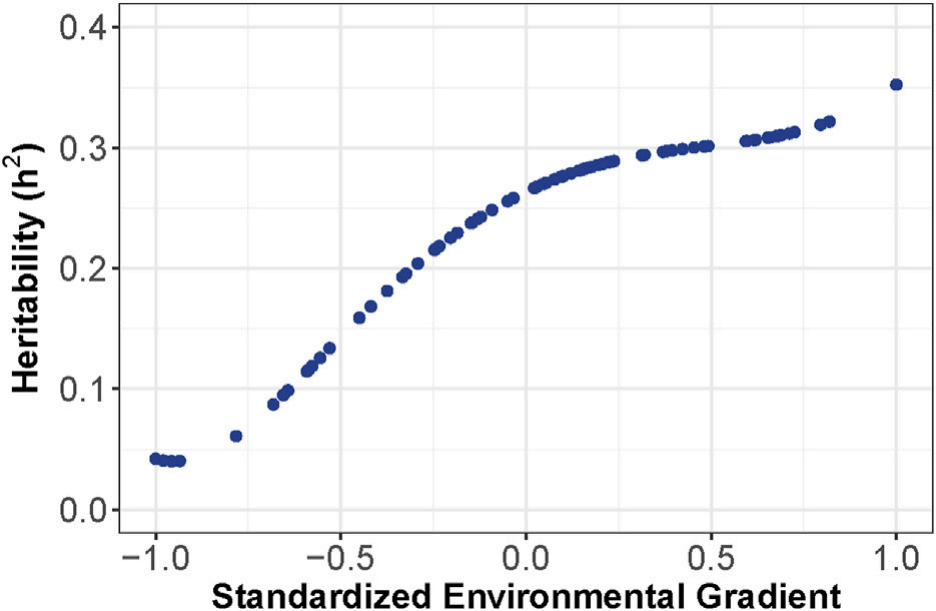
Narrow-sense heritability of yield in each of the 86 environments represented as a gradient from −1 (lowest-yielding environment) to 1 (highest-yielding environment).

A summary of the RNM comparison is included in Table 2. The models compared included linear and polynomial models with either homogeneous or heterogeneous residual variance. The model with the best fit based on AIC was the 2nd order Legendre orthogonal polynomial with a heterogeneous residual variance. Variance components were estimated in the polynomial model with a heterogeneous residual variance. Narrow-sense heritability was inconsistent across the 86 environments with a range from 0.04 to 0.35 (Figure 2). Heritability was lowest in low-yielding environments with an increased heritability as the EG increased but plateaued at a heritability of about 0.3 in most environments.

**TABLE 2:** Comparison of the four models tested in this study using AIC. Model selected based on the lowest AIC value is in bold.

Model	Residual	AIC^a^
1st Order Legendre Polynomial	Homogeneous	136,114.9
1st Order Legendre Polynomial	Heterogeneous	136,113.92
2nd Order Legendre Polynomial	Homogeneous	135,927.43
**2nd Order Legendre Polynomial**	**Heterogeneous**	**135,922.88**

^a^ Akaike Information Criterion.

**FIGURE 3:**
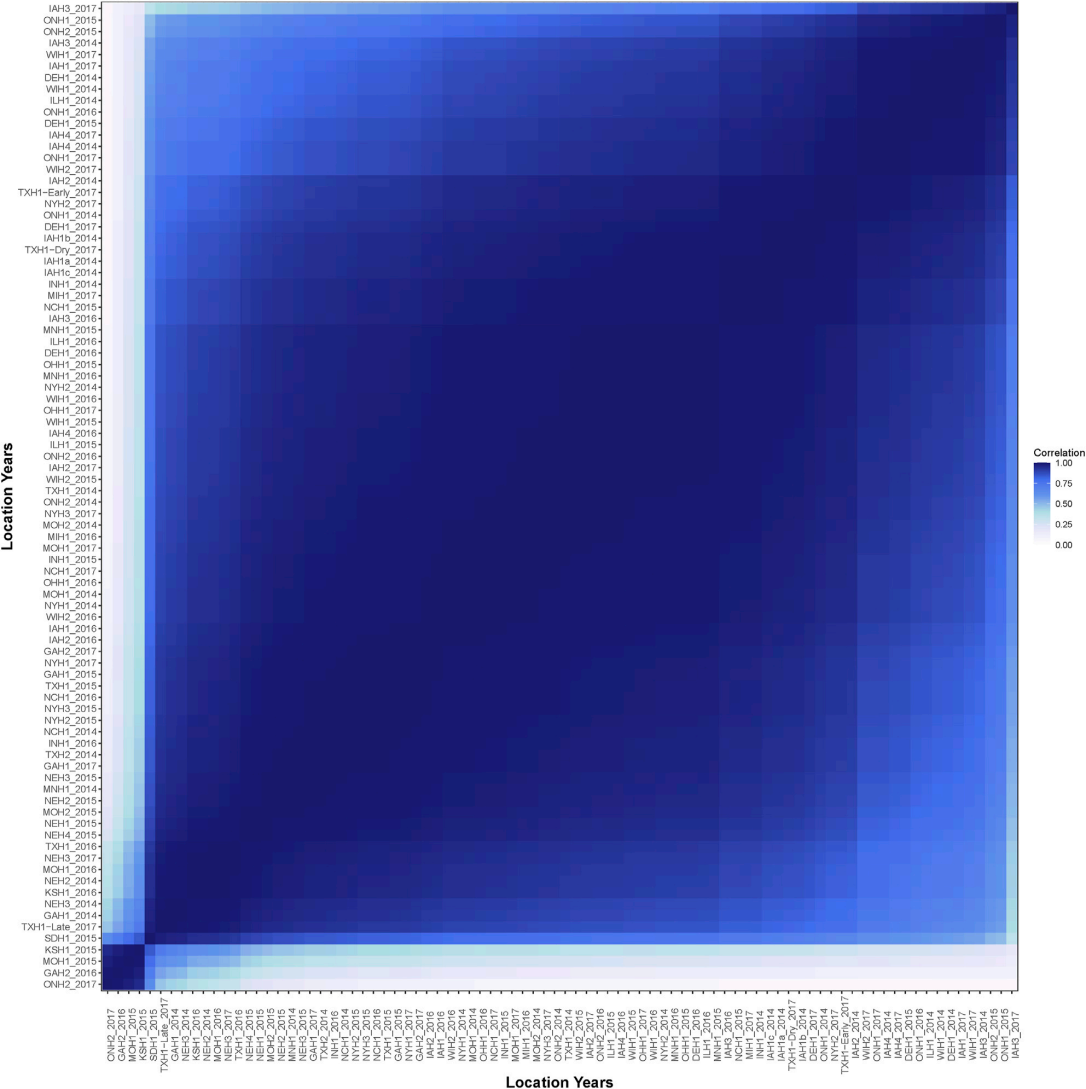
Estimated genetic correlation of yield across the 86 environments.

The average genetic correlation across the 86 environments was 0.87 (Figure 3). No environment had a negative genetic correlation with another environment. The environments ONH2_2017, GAH2_2016, MOH1_2015, and KSH1_2015 were highly correlated with each other, but had a reduced correlation to all other environments. Generally, environments adjacent on the EG had a high genetic correlation and it decreased as the gap between the environments increased.

### 3.2 Genome-wide association study

**FIGURE 4:**
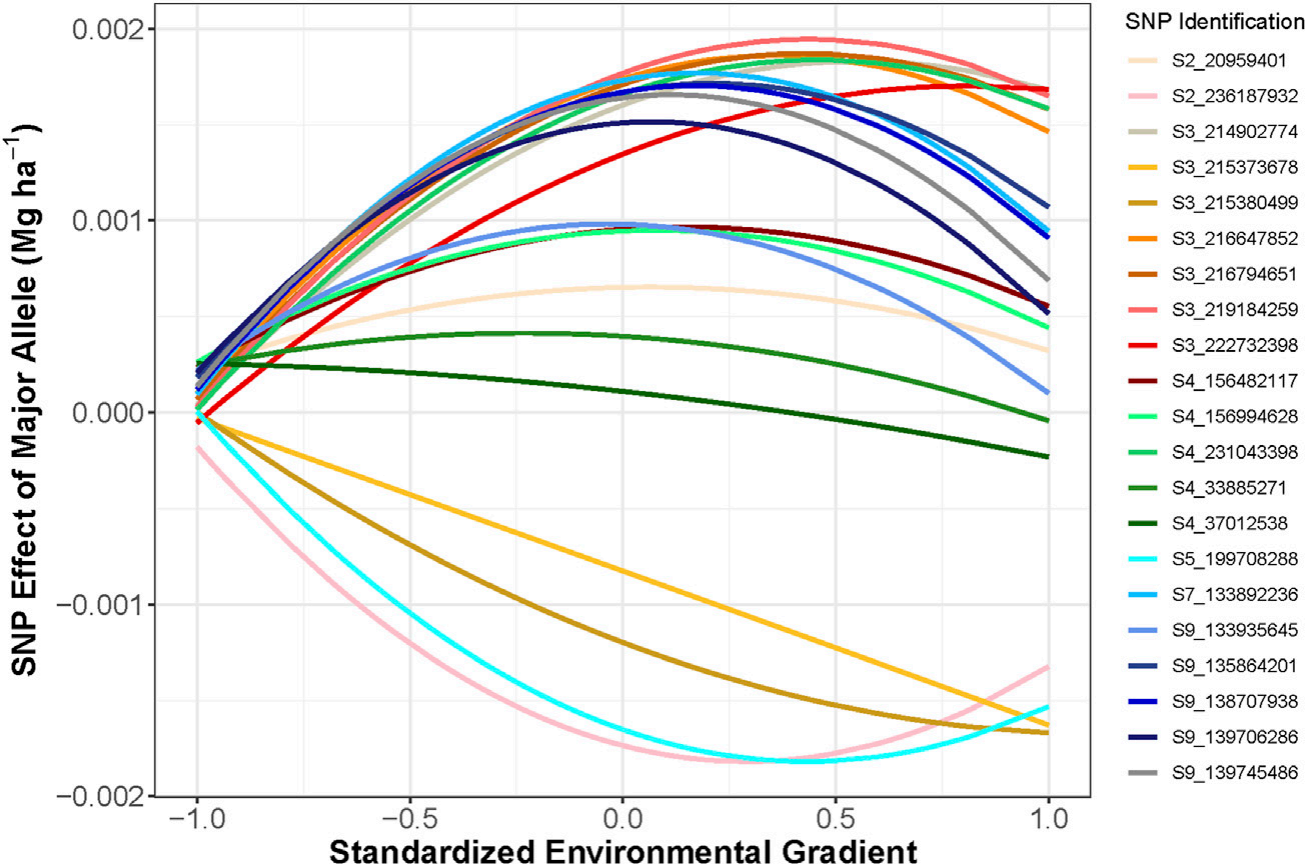
**S**ingle nucleotide polymorphism (SNP) effect of the major allele for the 21 selected SNPs. Negative SNP effect indicated that the minor allele had a positive effect on improving grain yield. Color was used to differentiate each SNP.

The top five SNPs were selected based on the magnitude of effect on grain yield in each environment and not in LD (*r* = 0.2) with any other selected SNP. Twenty-one SNPs were selected, which indicated that many environments had overlapping highest-effect SNPs (Figure 4). SNP effect magnitude was often greater in higher-yielding environments and the SNP effect was often consistent in environments adjacent on the EG. The favorable alleles of selected SNPs in environment ONH2_2017 were found to have a negative impact in many other environments. These results indicated that both magnitude and sign of SNP effects were dependent on environment. Candidate genes were explored for these 21 selected SNPs. Seventeen of the 21 SNPs were within ± 1 kb of annotated gene models (Table 3).

**TABLE 3:** Single nucleotide polymorphisms (SNPs) selected from each environment.

SNP_ID	Candidate genes	Annotation	Previous publications
S2_20959401	*Zm00001d002774*, *GRMZM5G859526, AC212835.3_FG004*	Uncharacterized protein	Robbins (2017)
S2_236187932	*Zm00001d007962*, *GRMZM2G173882*	Myb-like DNA-binding domain, SHAQKYF class family protein	Guo et al., 2022
S3_214902774	*Zm00001d044083*, *GRMZM2G126260*	Auxin efflux carrier component	Han et al., 2020; Yue et al., 2021
S3_215373678	*Zm00001d044093*, *AC211319.3_FG001*, *GRMZM2G047961*, *AC211319.3_FG002*	CASP-like protein 4U1	*—*
S3_215380499	*Zm00001d044094*, *GRMZM2G048010*	Exostosin family protein	Tan et al., 2018
S3_216647852	*Zm00001d044139*, *GRMZM2G464976*	Uncharacterized protein	Monir and Zhu 2018
S3_216794651	*Zm00001d044142*, *GRMZM2G364528*	BHLH domain-containing protein	Wang et al. (2016)
S3_219184259	*Zm00001d044242*, *GRMZM2G081816*	Transcription factor bHLH87	Vendramin et al. (2020); Rodríguez-Gómez et al. (2022)
S3_222732398	*Zm00001d044379*, *GRMZM2G055578, GRMZM5G837381*, *GRMZM2G528502*	CASC3/Barentsz eIF4AIII binding	*—*
S4_33885271	*—*	*—*	*—*
S4_37012538	*Zm00001d049648*, *GRMZM2G302160*	Protein STICHEL-like 2	*—*
S4_156482117	*Zm00001eb428040*, *GRMZM2G149178*	Histone H4	Qi et al. (2016)
S4_156994628	*Zm00001d051502*, *GRMZM2G081310*	Calcium dependent protein kinase7	Zhang et al. (2022)
S4_231043398	*Zm00001d053603*, *GRMZM2G343139*	ABC transporter A family member 7	Cao et al. (2022)
S5_199708288	*—*	*—*	*—*
S7_133892236	*—*	*—*	*—*
S9_133935645	*Zm00001d047587*, *GRMZM2G426964*, *GRMZM2G056099*	Glucose-6-phosphate 1-dehydrogenase, 1.1.1.49	*—*
S9_135864201	*—*	*—*	*—*
S9_138707938	*Zm00001d047764*, *GRMZM2G011071*	Uncharacterized protein	*—*
S9_139706286	*Zm00001d047807*, *GRMZM2G103247*	protein-serine/threonine phosphatase, 3.1.3.16	Willcox et al. (2022)
S9_139745486	*Zm00001d047808*, *GRMZM2G063151, GRMZM2G569643*	Initiator binding protein1	Xu et al. (2016)

Candidate genes were explored that were ±1 kb from the selected SNPs using the Maize B73 v4 reference genome in https://www.maizegdb.org and the annotations are from UniProt database (https://www.uniprot.org/). A literature review was performed to find relevant publications that have previously identified these candidate genes.

SNPs S2_236187932, S3_215373678, S3_215380499, and S5_199708288 had favorable minor alleles in the population. Within each environment, hybrids were grouped by their SNP state to determine significant differences in the yield of these SNP states. Hybrids at differing SNP states for S2_236187932 was considered in 37 environments. Significant differences between the three SNP states were observed in 16 of the 37 environments, though in 13 of the 16 environments the hybrids homozygous for the major alleles were favorable which indicated conflicting results.

Eighteen of the 38 environments for S3_215373678 resulted in a significant improvement in grain yield in either the homozygous minor allele or heterozygous hybrids compared with homozygous major allele hybrids. The greatest difference for S3_215373678 was in IAH1b_2014 with average yields of 10.78, 10.98 and 9.3 Mg ha^−1^ for the homozygous minor allele, heterozygous, and homozygous major allele hybrids.

Twenty-four of the 41 comparisons for S3_215380499 had hybrids with grain yields that were significantly improved in either the homozygous minor allele or heterozygous hybrids compared with homozygous major allele hybrids. Sixteen of these environments resulted in significant differences between the homozygous minor and homozygous major allele hybrids where the minor allele was favorable by more than 0.5 Mg ha^−1^.

Seventeen of the 45 environments evaluated for S5_199708288 had a significant decrease in average yield of the hybrids homozygous for the major allele compared with the other SNP states. The greatest difference was in environment ONH2_2014 where grain yields of 9.59, 9.68, and 7.72 Mg ha^−1^ were observed for the hybrids homozygous for the minor allele, heterozygous, and homozygous for the major allele, respectively. In this environment, the homozygous major allele hybrids had significantly lower yield than the hybrids with the other SNPs states.

### 3.3 Genomic prediction of breeding values

In CV0-Predicted EG and CV00-Predicted EG, the merit of a given environment was determined using a machine learning model that incorporated weather and soil data specific to each environment (Table 1). Prediction accuracy of this model to predict the estimated value of the environments was 0.38 (Supplementary Figures S4, S5). Thus, certain environments were reranked when weather and soil variables were used to predict merit.

GEBVs were compared to single-environment BLUP that did not utilize genotype or environment data and the whole-dataset model where all data was used in training described in Eq. 3. Baseline GBLUP models were compared with those models that incorporated RNM. The average prediction accuracy of the CV0 models were 0.37, 0.34, and 0.32 for CV0-GBLUP, CV0-Predicted EG, and CV0-Known EG, respectively. Average Bias of −0.42, −0.34, and −0.37 was observed for CV0-GBLUP, CV0-Predicted EG, and CV0-Known EG. The CV00 scheme saw a reduced prediction accuracy in comparison to all CV0 models. For CV00 models, average prediction accuracy was 0.20 for CV00-GBLUP and 0.21 for CV00-Predicted EG. Average bias was −0.60 in CV00-GBLUP and −0.52 in CV00-Predicted EG.

**FIGURE 5:**
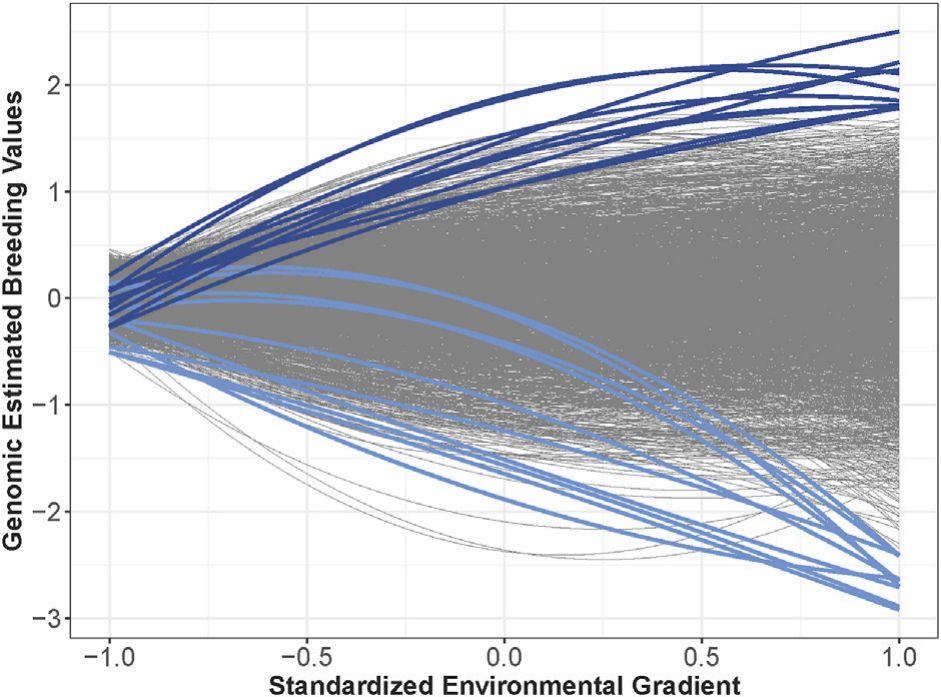
Genomic estimated breeding values for all 2,126 hybrids across the environmental gradient. Certain hybrids were colored to illustrate genotype-by-environment interactions with some hybrids value increased and others decreased in the higher-yielding environments.

GEBVs were compared to their counterparts where all hybrids in all environments were used for model development (Figure 5). Prediction accuracy of CV0-GBLUP ranged from −0.13 to −0.95 with an average of 0.72. An increase in prediction accuracy was observed in using the RNM model with either a predicted EG or known EG in comparison to the GBLUP model. Prediction accuracy ranged from −0.14 to 0.99 with an average of 0.80 in CV0-Predicted EG and 0.08 to 0.98 with an average of 0.81 in CV0-Known EG. Average bias across the environments was −0.38, −0.15, and −0.15 in CV0-GBLUP, CV0-Predicted EG, and CV0-Known EG, respectively. Reduced prediction accuracy was again observed when comparing CV00 models to CV0 models when predicting the whole-dataset GEBV for each environment. CV00-GBLUP had a range in prediction accuracy from 0.10 to 0.78 with an average of 0.57. CV00-Predicted EG had a range in prediction accuracy from 0.05 to 0.86 with an average prediction accuracy of 0.60. Average bias improved in CV00-Predicted EG (−0.21) when compared to CV00-GBLUP (−0.37).

## 4 Discussion

Hybrid selection in maize is largely determined based on grain yield performance. Nevertheless, grain yield is affected by complex GEI indicating that a single hybrid may not maximize yield in all environments. Reaction norm models have been used to model GEI across a continuum of weather and soil variables used to characterize environments (Jarquín et al., 2014; Westheus et al., 2021). Jarquín et al. (2014) and Westheus et al. (2021) found models that incorporated interaction effects between environmental covariates and genetics were more predictive than models that did not include GEI effects. Nevertheless, only a limited proportion of across-environment variation was explained using the environmental covariates (Jarquín et al., 2014). In this study, environments were organized based on the estimated merit of the environment with the assumption that a given hybrid will have similar performance in environments with similar merit. The RNM was used to evaluate environment-dependent SNP effects and to estimate the GEBV of all hybrids along a gradient of environmental merit. Genomic prediction was performed to assess the prediction accuracy of this model.

### 4.1 Heritability and genetic correlation of environments

The heritability estimates were lowest in the low-yielding environments with a range from 0.04 to 0.35 (Figure 2). The increased heritability in the higher-yielding environments indicated that selection in these environments could be more beneficial to improve genetic gain than selection in lower-yielding environments with a reduced heritability. One approach to improve heritability of lower-yielding, stressed environments is to perform managed-stress trials where trial uniformity and repeatability are generally improved (Cooper et al., 2014). The average genetic correlation was 0.87 across the 86 environments in this study. Hybrid performance could be expected to be more similar in environments with a high genetic correlation than in less correlated environments. Most of the environments had a large, positive genetic correlation; however, four of the environments were unrelated to the other 82 environments but were highly correlated amongst themselves (Figure 4). Another method to assess the genetic correlation of yield across environments could be using a factor analytic model. In this approach, genetic effects are divided between environment-independent and environment-dependent effects to model GEI. Rogers et al. (2021) used a factor analytic model and reported high genetic correlation across many of these same environments from 2014 to 2016. However, their genetic correlation was often reduced compared to what was observed in this study. While genetic correlation was high in this study, GEI was observed as both change in rank and magnitude across environments influencing hybrid performance (Figure 5).

The lowest-yielding, least-heritable environments with a poor genetic correlation to other environments were ONH2_2017, GAH2_2016, MOH1_2015, and KSH1_2015. Environmental conditions limited the genetic variability of these environments (Supplementary Figure S6). Average days to anthesis in ONH2_2017 was 38 days while the average among other environments was 70 days after planting. Thus, it is likely that growing degree days limited the yield potential of higher-yielding hybrids in this environment. Maximum daily temperature in GAH2_2016 reached between 35°C and 40°C throughout the window surrounding flowering and was among the hottest environments throughout the growing season. GAH2_2016 appeared to be a heat-stress environment where higher-yielding, vulnerable hybrids did not reach their yield potential due to the stress. Within-field spatial variability where the environment had a greater impact on yield performance of a given plot than genetics was a key factor in MOH1_2015 and KSH1_2015.

### 4.2 Candidate genes associated with yield across environments

SNP effects were observed across a gradient of environmental conditions. Due to GEI, it was expected that SNP effects could be variable across environments (Millet et al., 2016; Yuan et al., 2019). In this study, different SNPs were selected based on their magnitude of effect in high- vs low-yielding environments suggesting that SNP effects were environment dependent. In total, 21 SNPs were selected in this study as having a high magnitude of effect in at least one environment (Figure 4). Sixteen of these SNPs were persistent and were selected in at least two environments and 11 of the SNPs were selected in more than 10 environments.

Homozygous minor alleles conferring greater yield performance were further explored as these represent areas that could be beneficial for further population improvement. Out of the 21 selected SNPs examined, four SNPs (S2_236187932, S3_215373678, S3_215380499, and S5_199708288) were shown to have a positive effect for the minor allele. S5_199708288 was the only SNP not within 1 kb of an annotated gene models. S2_236187932 was within 1 kb of candidate gene *Zm00001d007962*. Guo et al. (2022) previously identified this candidate gene as a potential transcription factor involved in regulating nitrogen metabolism and could be a useful adaptation in low nitrogen conditions. S3_215373678 was within 1 kb of candidate gene *Zm00001d044093* and had an annotated function of a CASP-like protein. S3_215380499 was a SNP within 1 kb of *Zm00001d044094* which Tan et al. (2018) found to be differentially expressed in tassels under drought-stressed and normal conditions.

Grain yield of hybrids from varying SNP states were compared to verify the advantage of the minor allele for these four SNPs. Grain yield was often improved in the homozygous minor allele or heterozygous hybrids for S3_215373678, S3_215380499, and S5_199708288 compared to homozygous major allele hybrids. For comparison of yield performance differences between hybrids with the minor allele and the major allele, we selected environments where there were at least ten hybrids representing the minor allele and ten hybrids with the major allele. The average yield increase was 0.21 Mg ha^−1^ across 57 environments for S3_215373678, 0.42 Mg ha^−1^ across 64 environments for S3_215380499, and 0.21 Mg ha^−1^ across 68 environments for S5_199708288. Thus, these SNPs could be important selection targets for population improvement.

Among the selected SNPs in this study, multiple candidate genes have previously been identified as having an association to stress-tolerance related traits. Candidate gene, *GRMZM2G126260*, was found by Han et al. (2020) and Yue et al. (2022) to be involved in deep-sowing tolerance impacting a genotypes ability to respond to drought stress. The annotated function of candidate gene *Zm00001d051502* was a calcium dependent protein kinase which contributes to the growth and development, as well as the abiotic and biotic stress tolerance of a plant (Kong et al., 2013). *Zm00001d044242* was identified as a transcription factor involved in biotic (Rodríguez-Gómez et al., 2022) and abiotic (Vendramin et al., 2020) stress tolerance. In summary, many of the candidate genes found in this study appear to be adaptations related to stress tolerance.

Since many of the candidate genes are associated with stress tolerance, it could be expected that hybrids with differing alleles at these SNPs could have different yields especially in stress-induced, low-yielding environment. Significant differences between hybrids with differing alleles were more common in the medium- to higher-yielding environments than in the low-yielding environments. These results suggest that alleles conferring greater stress tolerance could be beneficial across a range of environmental conditions.

### 4.3 Comparison of genomic prediction methods

Accurately predicting hybrid performance without growing field-experiments offers a major opportunity to improve genetic gain in maize. As prior knowledge and the GWAS analyses indicate grain yield in these environments is polygenic in nature, genomic prediction was assessed in this study. A standard GBLUP model was used for comparison to understand the value of the RNM. The GBLUP model outperformed the RNM models in prediction accuracy when GEBV were compared to single-environment BLUPs. Average prediction accuracy was 0.37, 0.34, and 0.32 in CV0-GBLUP, CV0-Predicted EG, and CV0-Known EG, respectively. Similar prediction accuracies were observed from CV00-GBLUP (*r* = 0.20) and CV00-Predicted EG (*r* = 0.21). When GEBV from GP were compared to whole-dataset GEBV, RNM models outperformed GBLUP. Average prediction accuracy was 0.72, 0.80, and 0.81 in CV0-GBLUP, CV0-Predicted EG, and CV0-Known EG. CV00-Predicted EG (*r* = 0.60) had a higher average prediction accuracy than CV00-GBLUP (*r* = 0.57). In all of these scenarios, the GBLUP models consistently had a bias further from 0 than the RNM models. This could be due to the lack of GEI captured in the GBLUP model.

Prediction accuracy was greater where tested hybrids were grown in an untested year (CV0-Predicted EG; *r* = 0.34) compared to untested hybrids in an untested year (CV00-Predicted EG; *r* = 0.21) (Table 4). A benefit of the publicly available Genomes to Fields initiative dataset is that results from different studies that have used this dataset can be easily compared. Rogers and Holland (2022) performed across-year prediction, like CV0-Predicted EG, where GEI was described using marker-by-environment descriptors. A principal component analysis was used on either the marker or environmental data for dimension reduction. Models with just the main effects of markers and environmental covariates outperformed these GEI models with an average prediction accuracy of 0.29. Westhues et al. (2021) performed genomic prediction using various linear random effect models and machine learning based methods. Their prediction accuracy ranged from 0.31 to 0.42 depending on the model with xgboost performing better than the other model types.

**TABLE 4:** Genomic prediction (GP) accuracy and bias across 86 environments.

	BLUP					Whole-dataset				
Cross validation	CV0			CV00		CV0			CV00	
Model	GBLUP	Predicted EG	Known EG	GBLUP	Predicted EG	GBLUP	Predicted EG	Known EG	GBLUP	Predicted EG
Mean	0.37	0.34	0.32	0.20	0.21	0.72	0.80	0.81	0.57	0.60
Minimum	−0.36	−0.40	−0.21	−0.16	−0.24	−0.13	−0.14	0.08	0.10	0.05
Maximum	0.77	0.70	0.68	0.52	0.54	0.95	0.99	0.98	0.78	0.86
Standard Deviation	0.20	0.18	0.18	0.14	0.15	0.20	0.20	0.17	0.16	0.16
Bias	−0.42	−0.34	−0.37	−0.60	−0.52	−0.38	−0.15	−0.15	−0.37	−0.21

Genomic estimated breeding values from GP were either associated to single-environment BLUPs or to the estimated GEBV of the whole-dataset model where all data was used in training. CV0 allowed for hybrids evaluated in the training set in another environment to be in the testing set, while CV00 only considered unobserved hybrids in the testing set. Prediction accuracy was assessed using Pearson’s correlation for each environment and summary statistics of mean, minimum, maximum, and standard deviation are provided. Bias (*b*_*1*_) was evaluated for each environment using a linear regression model (BLUP = *b*_*0*_ *+ b*_1_*GEBV_*testing*_) or (GEBV_*whole*_ = *b*_*0*_ *+ b*_1_*GEBV_*testing*_). Bias was centered around 0 by subtracting one from the regression coefficient.

In this study, the within-environment BLUP prediction accuracy varied substantially across environments with ranges from −0.40 to 0.70 in CV0-Predicted EG and from −0.24 to 0.54 in CV00-Predicted EG. As such, a few environments were negatively impacting the average prediction accuracy. Prediction accuracy in MNH1_2014 was −0.40 in CV0-Predicted EG and was far worse than the next worse prediction accuracy of −0.02 in KSH1_2015. Westhues et al. (2021) previously found performance in this environment to be difficult to predict (*r* = −0.14); however, including environment data in the model in their study improved the prediction accuracy of this environment (*r* = 0.42). Other environments in their study performed worse when environmental data was included which indicated there was no ideal model for prediction in all environments.

The merit of an environment in this study was estimated as the fixed effect of environment in a BLUP model to mitigate differences in average yield performance due to different hybrids evaluated in different environments. Where yield is unknown in an environment, such as in the testing set of genomic prediction, it is possible to use environmental characteristics (latitude, longitude, weather, soil, etc.) to estimate the merit of a given environment. In this study, a Random Forest model was trained to associate the environmental data from 3 years of data to their known environmental merits and was used to predict environmental merit of the other year (that corresponded to the testing dataset of genomic prediction). The correlation between known and predicted environmental merit was *r* = 0.38. This model has since been applied in the 2022 G2F yield prediction contest using data from 2014 to 2021, and an increased environmental merit prediction accuracy was observed due to the increase in the number of environments observed.

Since there was a discrepancy between known and predicted environmental merit in this study, there was reranking between environments on the environmental gradient that could have impacted prediction accuracy. Genomic prediction was assessed with the known environmental merit to see if this would improve prediction accuracy. Average GP accuracy with the known environmental merit (CV0-Known EG) was 0.32 with a range from −0.21 to 0.68 and did not improve prediction accuracy over the GP model with predicted environmental merit (CV0-Predicted EG). This lack of change is likely due to the high genetic correlation across environments in this study. As such prediction accuracy was less impacted by placement on the EG.

Often GP accuracy is compared to observed yield of hybrids in a given environment from a BLUP model. As within-environment BLUP models do not account for the relationship between environments and occasionally use an identity matrix to describe the relationship between hybrids, it is likely that these are not the most accurate representation of a hybrids true value. In this study, GEBVs from GP were compared to a whole-dataset model where phenotypic and genotypic data of all hybrids in all environments was modeled. Prediction accuracy of CV0-Predicted EG and CV00-Predicted EG were 0.80 (range: −0.14–0.99) and 0.60 (range: 0.05–0.86) and was greatly improved in comparison to the prediction accuracy of the BLUP model.

### 4.4 Implications, limitations, and future work

The results of this study indicate the value of RNM to better understand GEI and predict hybrid yield performance. Twenty-one SNPs with associated candidate genes were found to be associated to yield in environments across the EG, and many of the candidate genes were previously reported as stress adaptations. Genomic prediction was performed with an increased correlation to models that incorporated genomic and environmental relationships than within-environment BLUP models. While yield was used to describe environmental performance in this study, this strategy is potentially limited in breeding for stress adaptation. Cairns et al. (2013) found hybrid performance in one type of stress is not indicative to performance in another type of stress. As such, in another trial where the genetic correlation of yield across environments is lower, the methodology presented in this paper might have a decrease in performance. Genomic prediction was only performed in this study for hybrids with phenotypic data. Nevertheless, this dataset, and more recent years of the G2F initiative, could be used as a training dataset to evaluate all possible hybrid combinations with genomic information to greatly expand the number of hybrids evaluated.

## 5 Conclusion

Large-scale genomic sequencing capabilities enables genome-wide association studies and genomic prediction with the benefits of assessing hybrid merit and minimizing the phenotyping cost of multi-environment trials. A reaction norm model was applied to model hybrid yield across a range of environmental conditions. Heritability was improved in the higher-yielding environments which indicated that hybrid performance in the low-yielding environments was relatively more dependent on the environment. Genome-wide association study was used to estimate SNP effect, and many candidate genes were found to be associated with stress tolerance in previous studies. Multiple genomic prediction scenarios were performed where prediction accuracy was greater when hybrids had previously been tested than when untested hybrids were evaluated in an untested environment. Reaction norm models outperformed GBLUP when compared to GEBV derived models that utilized the whole-dataset. This approach could be useful for improving genetic gain in maize by increasing the number of hybrids and environments evaluated while limiting cost associated with phenotypic evaluation in multi-environment trials.

## Data Availability

The datasets presented in this study can be found in online repositories. The names of the repository/repositories and accession number(s) can be found in the article/Supplementary Material.
