# Supplementary material for: Genomic prediction and association mapping of maize grain yield in multi-environment trials based on reaction norm models
Source: Front Genet. 2023 Aug 31;14:1221751. doi: 10.3389/fgene.2023.1221751 (PMC10501150; doi:10.3389/fgene.2023.1221751)
Supplement: Supplementary file 2 [file DataSheet1.docx]

Supplementary Material

**Genomic Prediction and Association Mapping of Maize Grain Yield in Multi-environment Trials Based on Reaction Norm Models**

Seth A. Tolley^1^, Luiz F. Brito^2^, Diane R. Wang^1^, Mitchell R. Tuinstra^1,*^

*** Correspondence:** Mitchell Tuinstra: mtuinstr@purdue.edu

# Supplementary Figures and Tables


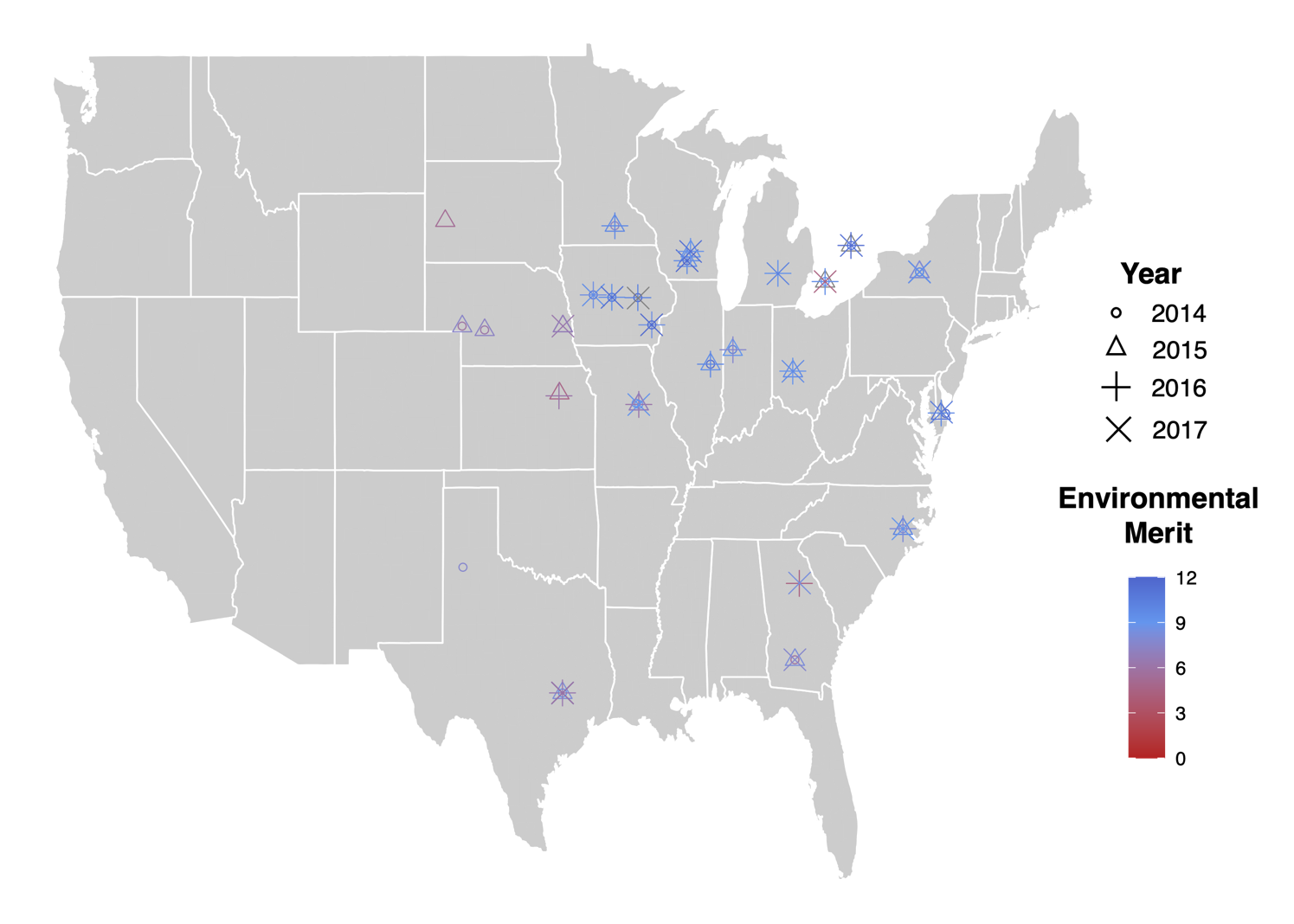


**Supplemental Figure 1.** Map of the 86 environments (location-year) used in this study where different years are illustrated using shapes of different size and the environmental merit of an environment is displayed as the color.

**Supplemental Figure 2.** Comparison of within-environment repeatability for the 105 environments with and without a spatial correction. The 97 environments that are above the 1-1 line had an improved repeatability with a spatial correction.

**Supplemental Figure 3.** Linkage disequilibrium (LD) of the 2,126 hybrids used in this study. An LD of 0.2 (indicated by the horizontal red dashed line) was reached at about 1 kb in each chromosome. The black line represented the average of the 10 chromosomes.

**Supplemental Figure 4.** Prediction accuracy of the environmental merit from a machine learning model using weather, soil, and geographic information.

**Supplemental Figure 5.** Variable importance from machine learning model to predict environmental merit using weather, soil, and geographic information. The number at the end of the variable string indicates the month in the year.

**Supplemental Figure 6.** Growing degree days (GDD) (A) and maximum daily temperature (B) across the growing season for each of the 86 environments. The four lowest-yielding environments are given in different colors to identify if there were noticeable patterns that made these environments low yielding.
